# The Effects of Mutual Interaction of Orexin-A and Glucagon-Like Peptide-1 on Reflex Swallowing Induced by SLN Afferents in Rats

**DOI:** 10.3390/ijms21124422

**Published:** 2020-06-22

**Authors:** Motoi Kobashi, Yuichi Shimatani, Masako Fujita, Yoshihiro Mitoh, Ryusuke Yoshida, Ryuji Matsuo

**Affiliations:** 1 Department of Oral Physiology, Okayama University Graduate School of Medicine, Dentistry and Pharmaceutical Sciences, Okayama 700-8525, Japan; mfujita@cc.okayama-u.ac.jp (M.F.); ymitoh@md.okayama-u.ac.jp (Y.M.); yoshida.ryusuke@okayama-u.ac.jp (R.Y.); mkobashi@cc.okayama-u.ac.jp (R.M.); 2 Department of Medical Engineering, Faculty of Science and Engineering, Tokyo City University, Tokyo 158-8557, Japan; yshimata@tcu.ac.jp

**Keywords:** GLP-1, orexin, SB334867, swallowing, NTS, rats

## Abstract

(1) Background: Our previous studies revealed that orexin-A, an appetite-increasing peptide, suppressed reflex swallowing via the commissural part of the nucleus tractus solitarius (cNTS), and that glucagon-like peptide-1 (GLP-1), an appetite-reducing peptide, also suppressed reflex swallowing via the medial nucleus of the NTS (mNTS). In this study, we examined the mutual interaction between orexin-A and GLP-1 in reflex swallowing. (2) Methods: Sprague–Dawley rats under urethane–chloralose anesthesia were used. Swallowing was induced by electrical stimulation of the superior laryngeal nerve (SLN) and was identified by the electromyographic (EMG) signals obtained from the mylohyoid muscle. (3) Results: The injection of GLP-1 (20 pmol) into the mNTS reduced the swallowing frequency and extended the latency of the first swallow. These suppressive effects of GLP-1 were not observed after the fourth ventricular administration of orexin-A. After the injection of an orexin-1 receptor antagonist (SB334867) into the cNTS, an ineffective dose of GLP-1 (6 pmol) into the mNTS suppressed reflex swallowing. Similarly, the suppressive effects of orexin-A (1 nmol) were not observed after the injection of GLP-1 (6 pmol) into the mNTS. After the administration of a GLP-1 receptor antagonist (exendin-4(5-39)), an ineffective dose of orexin-A (0.3 nmol) suppressed reflex swallowing. (4) Conclusions: The presence of reciprocal inhibitory connections between GLP-1 receptive neurons and orexin-A receptive neurons in the NTS was strongly suggested.

## 1. Introduction

Swallowing is an early step in feeding behavior to propel food into the stomach, and is caused by simultaneous mastication and gastric accommodation. Both mastication and gastric accommodation are altered by orexigenic substances [1,2,3]. Anorectic substances also change gastric motor function [4]. It is therefore inferred that orexigenic and/or anorectic substances affect swallowing and other digestive functions. Our previous study revealed that appetite-enhancing peptides, such as ghrelin and orexin/hypocretin, suppressed reflex swallowing induced by afferent stimulation of the superior laryngeal nerve (SLN) [5,6]. Our previous study and those of others demonstrated that the injection of a satiety peptide such as glucagon like peptide-1 (GLP-1), leptin, oxytocin, or nesfatin-1 into the rat dorsal medulla suppressed reflex swallowing induced by electrical stimulation of the SLN, similarly to appetite-enhancing peptides [7,8,9]. Thus, both appetite-increasing and -satiating substances similarly suppress reflex swallowing. The role of this paradoxical response is thought to be helpful in keeping the ability of reflex swallowing constant even after meals [9].

There are a number of appetite-related peptides. Some of these are known to interact with each other. Leptin inhibits neuropeptide Y/agouti-related peptide (NPY/AgRP) neurons and conversely excites pro-opiomelanocortin/cocain-and amphetamin-regulated transcript (POMC/CART) neurons [10]. The satiety peptide cholecystokinin (CCK) inhibits the action of ghrelin on food intake and vice versa [11]. Orexin also affects other appetite-regulating peptides. Orexin-A reversed the CCK-induced loss of appetite [12] and inhibited the effects of the satiated hormone amylin on meal size [13]. Furthermore, the anorexic effects of liraglutide, a GLP-1 receptor antagonist, may be caused by reduced orexin activity [14]. Thus, GLP-1 and orexin interact in relation to feeding. Orexins are synthesized mainly in the lateral hypothalamic area (LHA) [15,16]. Orexin-containing neurons are distributed widely in the brain, including the dorsal medulla [17], and are involved in the regulation of many motor functions associated with feeding [3,18,19]. GLP-1 released from the intestines activates sensory afferent neurons originating in the nodose ganglion, which may in turn activate neurons of the nucleus tractus solitarius (NTS) [20,21,22]. As histological studies demonstrated the presence of immunoreactive cells to GLP-1 in the NTS [23,24], brain-derived GLP-1 may affect food intake and feeding-related phenomena. Thus, the NTS is an important site of the action of GLP-1. These lines of evidence suggest that appetite-increasing signals descending from the LHA and peripheral (or lower brainstem) satiated signals interact in the dorsal medulla to alter feeding-related phenomena, including reflex swallowing.

The present study was designed to clarify whether GLP-1 and orexin-A interact in the control of reflex swallowing. First, how orexin-A affects the suppressive effects of GLP-1 on reflex swallowing was examined. Next, how GLP-1 affects the suppressive effects of orexin-A on reflex swallowing was examined.

## 2. Results

### 2.1. Orexin-A Suppresses the GLP-1 Response of Reflex Swallowing

**Figure 1:**
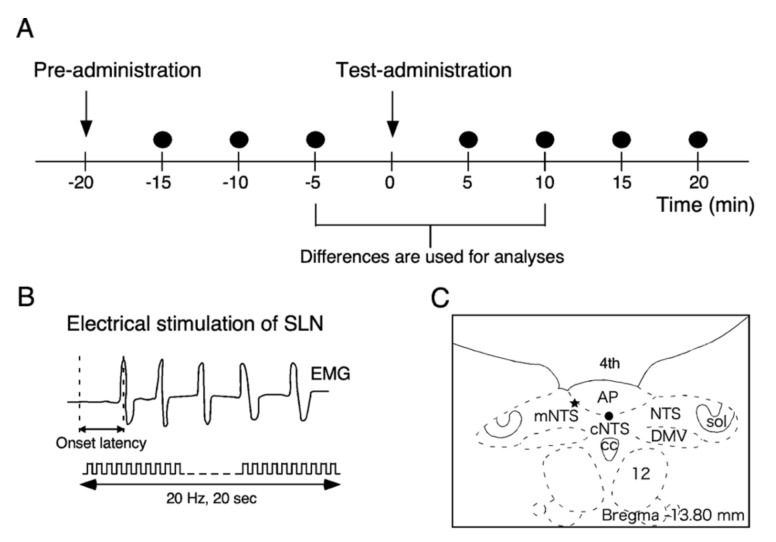
Schematic representation of the experimental protocol. (**A**) Time schedule of each experiment. Pre-administration of the drug was carried out 20 min before the test administration. Electrical stimulation of the superior laryngeal nerve (SLN) for 20 s was delivered every 5 min (filled circles). The differences between 5 min before the test administration and 10 min after were used as representative values. (**B**) Parameters used in data analyses. The number of swallows during electrical stimulation for 20 s was counted for every stimulus event (swallowing frequency). The time between the onset of stimulation and the peak of electromyographic (EMG) activity of the first swallow just after the onset of stimulation was measured and defined as latency. (**C**) Schematic representation of the tip locations of the pipette for drug injection. The tip of the pipette was located in the boundary between the area postrema (AP) and the commissural part of the nucleus tractus solitarius (cNTS) (filled circle) or in the boundary between the AP and the medial nucleus of the NTS (mNTS) (asterisk). In all other cases, the solution was dropped into the fourth ventricle (4th).

**Figure 2:**
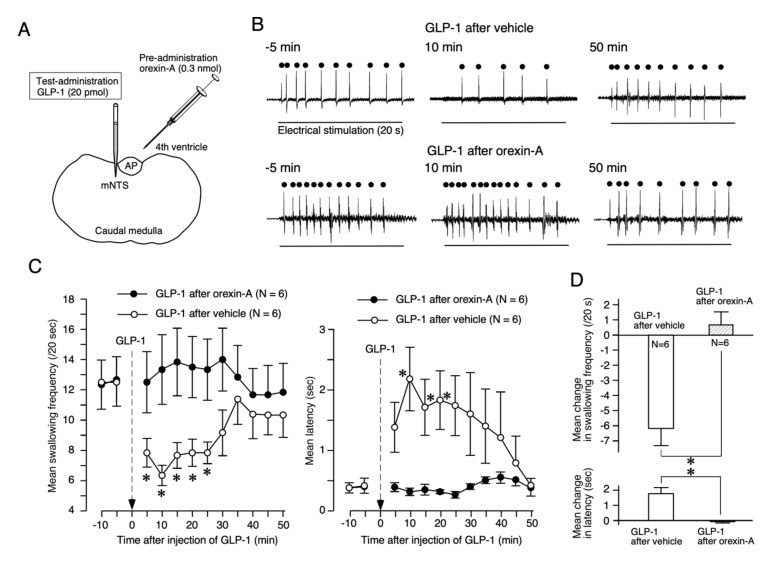
Pre-administration of orexin-A attenuated the suppressive effects of glucagon-like peptide-1 (GLP-1) on reflex swallowing. (**A**) Schematic representation of the sites of administration of drugs. Orexin-A was administered into the fourth ventricle 20 min before the administration of GLP-1. GLP-1 was injected into the mNTS. (**B**) EMG signals recorded from the mylohyoid muscle. Repetitive swallowing was observed during electrical stimulation of the SLN. The horizontal bar shows the period of stimulation. Filled circles indicate each swallow movement. The injection of GLP-1 into the mNTS (10 min in upper panel) suppressed reflex swallowing after vehicle administration. The suppressive effects of GLP-1 on reflex swallowing were not observed after the administration of orexin-A (10 min in lower panel). (**C**) The time course of the mean swallowing frequency (left) and mean latency (right) during electrical stimulation of the SLN is shown. Significant GLP-1 effects observed after vehicle administration (open circles) were not observed after the administration of orexin-A (filled circles). Asterisks indicate significant differences compared with the value 5 min before the injection of GLP-1 by Dunnett’s test. (**D**) The mean changes in swallowing frequency (upper) and latency (lower) induced by the injection of GLP-1 are presented. The pre-administration of orexin-A significantly reduced the suppressive effects of GLP-1. Asterisks indicate significant differences compared with the values obtained from the injection of GLP-1 after vehicle and after orexin-A.

The effects of orexin-A on the suppressive effects of GLP-1 were examined by the protocol shown in Figure 1. Prior to the injection of GLP-1 into the medial nucleus of the NTS (mNTS), orexin-A (0.3 nmol, 3 µL) or vehicle (3 µL) was administered into the fourth ventricle (Figure 2A). The injection of GLP-1 (20 pmol, 60 nL) into the mNTS after the administration of vehicle suppressed reflex swallowing, i.e., GLP-1 reduced the swallowing frequency and extended the latency, as shown in Figure 2B (upper traces). After the pre-administration of orexin-A, however, the suppressive effects of GLP-1 were not observed, as shown in Figure 2B (lower traces). The time course of the mean swallowing frequency (left) and mean latency (right) is shown in Figure 2C. After vehicle administration, injection of GLP-1 induced a significant change in swallowing frequency (F (5, 25) = 17.20, *p* < 0.0001). A significant change compared with 5 min before administration was observed at 5, 10, 15, 20, and 25 min after the injection of GLP-1. A significant change in latency (F (5, 25) = 4.90, *p* < 0.01) was also noted. A significant change compared with 5 min before administration was observed at 10, 15, and 20 min after the injection of GLP-1. After the administration of orexin-A, however, the injection of GLP-1 did not change the swallowing frequency (F (5, 25) = 1.20, Not Significant (NS)) or the latency (F (5, 25) = 1.00, NS). The mean changes in the swallowing frequency and latency after the administration of orexin-A were significantly smaller than those after administration of the vehicle (frequency: *t* = 4.67, *p* < 0.001; latency: *t* = 4.13, *p* < 0.01), as shown in Figure 2D.

### 2.2. An Orexin-1 Receptor Antagonist Facilitates the GLP-1 Response of Reflex Swallowing

**Figure 3:**
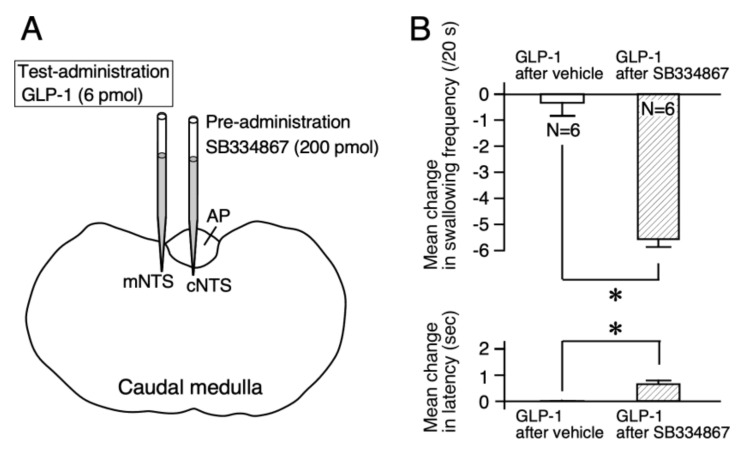
Pre-injection of an orexin-1 receptor antagonist (SB334867) into the cNTS facilitated the suppressive effects of GLP-1 on reflex swallowing. (**A**) Schematic representation of the sites of drug administration. SB334867 was injected into the cNTS 20 min before the injection of low-dose GLP-1 (6 pmol). GLP-1 was injected into the mNTS. (**B**) The mean changes in swallowing frequency (upper) and latency (lower) induced by the injection of GLP-1 after vehicle and those after SB334867 are presented. The injection of 6 pmol of GLP-1 after injection of the vehicle did not alter reflex swallowing (left). However, after the pre-injection of SB334867 into the cNTS, the suppressive effects of 6 pmol of GLP-1 were noted (right). Asterisks indicate significant differences compared with the values obtained from the injection of GLP-1 after vehicle and those after orexin-A.

For further confirmation of the effects of orexin-A on the GLP-1 response, blockade of orexin-1 receptors was examined. The low dose of GLP-1 (6 pmol, 60 nL) injected into the mNTS did not alter reflex swallowing [9]. We examined changes in the response to a non-effective dose of GLP-1 in reflex swallowing after administering an orexin-1 receptor antagonist. The orexin-1 receptor antagonist SB334867 (200 pmol, 60 nL) or vehicle was injected into the cNTS before the test injection of GLP-1 into the mNTS (Figure 3A). After injection of the vehicle, injection of the low dose of GLP-1 did not change the swallowing frequency (F (5, 25) = 2.02, NS) or the latency (F (5, 25) = 0.83, NS). After injection of SB334867, injection of GLP-1 reduced the swallowing frequency (F (5, 25) = 18.52, *p* < 0.0001) and extended the latency (F (5, 25) = 6.75, *p* < 0.0001). The mean changes in swallowing frequency and latency after the injection of SB334867 were significantly larger than those after injection of the vehicle (frequency: *t* = 7.90, *p* < 0.0001, latency: *t* = 3.58, *p* < 0.01), as shown in Figure 3B. The sole injection of SB334867 at 200 pmol into the cNTS did not change the swallowing frequency (F (5, 25) = 0.18, NS) or the latency (F (5, 25) = 0.44, NS).

### 2.3. GLP-1 Suppresses the Orexin-A Effects on Reflex Swallowing

**Figure 4:**
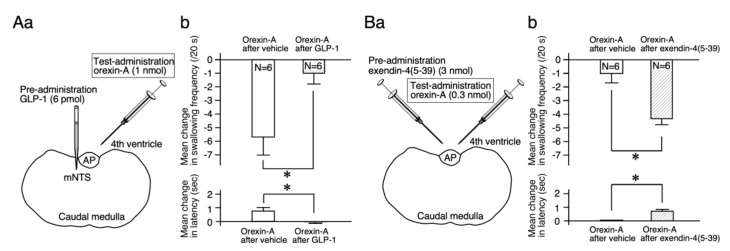
The effects of the pre-administration of GLP-1 or GLP-1 receptor antagonist (exendin-4(5-39)) on the suppressive effects of orexin-A on reflex swallowing. (**Aa**) Schematic representation of the sites of drug administration. GLP-1 (or vehicle) was injected into the mNTS 20 min before the test administration. Orexin-A was administered into the fourth ventricle (test administration). (**Ab**) The mean changes in swallowing frequency (upper) and latency (lower) induced by the administration of orexin-A after vehicle injection, and those after GLP-1 are presented. Orexin-A after vehicle injection suppressed reflex swallowing (left). After the injection of GLP-1 into the mNTS, the suppressive effects were not observed (right). Asterisks indicate significant differences compared with the values obtained from orexin-A after vehicle and those after GLP-1. (**Ba**) Schematic representation of the sites of drug administration. Exendin-4(5-39) or vehicle was administered into the fourth ventricle 20 min before the test administration. Low-dose orexin-A was also administered into the fourth ventricle (test administration). (**Bb**) The mean changes in swallowing frequency (upper) and latency (lower) induced by the administration of low-dose orexin-A (0.3 nmol) after vehicle administration and those after the administration of exendin-4(5-39) are presented. After vehicle administration, 0.3 nmol of orexin-A did not alter reflex swallowing (left). After the administration of exendin-4(5-39), suppressive effects were noted (right). Asterisks indicate significant differences compared with the values obtained from orexin-A after vehicle or exendin-4(5-39) administration.

Next, how GLP-1 affects the suppressive effects of orexin-A on reflex swallowing was examined. The effects of the administration of orexin-A into the fourth ventricle (1 nmol, 3 µL) after the injection of GLP-1 (6 pmol, 60 nL) or vehicle into the mNTS were examined (Figure 4Aa). After vehicle injection, the administration of orexin-A suppressed reflex swallowing. The swallowing frequency decreased (F (5, 25) = 5.46, *p* < 0.01) and the latency of the first swallow was extended (F (5, 25) = 3.35, *p* < 0.05). After injection of GLP-1 into the mNTS, the fourth ventricular administration of orexin-A did not change the swallowing frequency (F (5, 25) = 0.68, NS) or the latency (F (5, 25) = 1.13, NS). The mean changes in the swallowing frequency and latency after the injection of GLP-1 were significantly smaller than those after injection of the vehicle (frequency: *t* = 2.74, *p* < 0.05; *t* = 2.41, *p* < 0.05), as shown in Figure 4Ab.

### 2.4. A GLP-1 Receptor Antagonist Facilitates the Orexin-A Effects on Reflex Swallowing

For further confirmation of the effects of GLP-1 on the orexin response, blockade of the GLP-1 receptors was examined. The low dose of orexin-A (0.3 nmol) administered into the fourth ventricle did not alter reflex swallowing [6]. We examined changes in the response to a non-effective dose of orexin-A in reflex swallowing after administration of a GLP-1 receptor antagonist (exendin-4(5-39)). Exendin-4(5-39) (3 nmol, 3 µL) or vehicle was administered into the fourth ventricle before the test administration of orexin-A (3 nmol, 3 µL) into the fourth ventricle (Figure 4Ba). After vehicle administration, injection of a low dose of orexin-A did not change the swallowing frequency (F (5, 25) = 0.70, NS) or the latency (F (5, 25) = 0.98, NS). After the administration of exendin-4(5-39) into the fourth ventricle, the injection of orexin-A reduced the swallowing frequency (F (5, 25) = 3.63, *p* < 0.05) and extended the latency (F (5, 25) = 5.11, *p* < 0.01). The mean changes in the swallowing frequency and latency after the administration of exendin-4(5-39) were significantly larger than those after administration of the vehicle (frequency: *t* = 3.42, *p* < 0.01; latency: *t* = 3.01, *p* < 0.05), as shown in Figure 4Bb. The sole administration of exendin-4(5-39) (3 nmol, 3 µL) into the fourth ventricle did not change the swallowing frequency (F (5, 25) = 0.768, NS) or the latency (F (5, 25) = 0, NS).

## 3. Discussion

**Figure 5:**
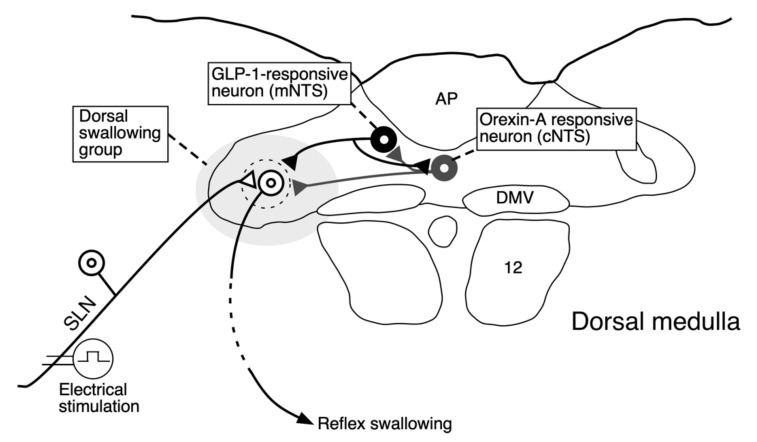
Schematic representation of a putative neural wiring diagram to induce the mutual interaction in reflex swallowing based on the present study and our previous studies [6,9]. The activation of GLP-1- or orexin-A responsive neurons resulted in suppression of reflex swallowing induced by electrical stimulation of the SLN. The suppressive effects of GLP-1 were eliminated by the activation of orexin-A-responsive neurons in the cNTS. The suppressive effects of orexin-A were eliminated by the activation of GLP-1-responsive neurons in the mNTS. As GLP-1 and orexin-A act in different regions in the NTS, reciprocal inhibition between GLP-1-receptive neurons and orexin-A-receptive neurons is hypothesized.

Our previous studies using the local administration of peptides, lesion of the area postrema (AP), and/or partial lesions of the NTS clarified that orexin-A and GLP-1 act on the cNTS and mNTS, respectively, to suppress reflex swallowing [6,9]. In the present study, the suppressive effects of GLP-1 injected into the mNTS on reflex swallowing were not observed after the administration of orexin-A (Figure 2). The dose of orexin-A used for pre-administration (0.3 nmol) does not significantly alter the swallowing reflex when administered alone [6]. As the suppressive effects of orexin-A on reflex swallowing are through the orexin-1 receptors in the cNTS [6], a small amount of orexin-1 receptor antagonist SB334867 was injected into the cNTS before the administration of GLP-1. As a result, the administration of a low dose of GLP-1, which did not affect reflex swallowing when administered alone, exerted suppressive effects on swallowing reflex after blockade of the orexin-1 receptors (Figure 3). These results suggest that orexin-A-receptive neurons in the cNTS were sending inhibitory signals to the GLP-1-receptive neurons in the mNTS (Figure 5). On the other hand, suppression of the swallowing reflex by the administration of 1 nmol of orexin-A was not observed after the injection of GLP-1 into the mNTS (Figure 4A). The dose of GLP-1 used for pre-injection (6 pmol) does not significantly alter the swallowing reflex when administered alone [9]. After administration of exendin-4(5-39), a GLP-1 receptor antagonist, a low dose of orexin-A (0.3 nmol), which does not affect the swallowing reflex when administered alone, suppressed the reflex swallowing (Figure 4B). Thus, a non-effective dose of orexin-A suppressed the reflex swallowing after blockade of the GLP-1 receptors. These results suggest that GLP-1-receptive neurons in the mNTS were sending inhibitory signals to the orexin-A-receptive neurons in the cNTS (Figure 5).

The present study suggests that there is mutual inhibition between the GLP-1-responsive neurons and orexin-A-responsive neurons (Figure 5). The most probable candidate transmitter for this mutual inhibition is gamma-aminobutyric acid (GABA). GABAergic neurons are widely distributed in the dorsal medulla, including the NTS [25]. The swallowing reflex is suppressed by the injection of GABA into the NTS [7]. Facilitatory effects of cannabinoids on reflex swallowing are caused by suppressing GABAergic neurons, termed disinhibition. [26]. Furthermore, GLP-1 affects the GABA-containing neurons in the caudal NTS [27]. The axons of orexin-containing neurons terminate adjacent to GABA- and/or GLP-1-containing neurons in the NTS [17]. The suppressive effects of orexin were suppressed by GABA antagonists [28]. It is therefore possible that GLP-1- and orexin-A-containing neurons mutually suppress reflex swallowing in the NTS via GABAergic neurons.

Numerous appetite-increasing and -satiating substances suppress reflex swallowing [5,6,7,9]. The swallowing reflex does not change much under normal conditions and sudden changes in swallowing conditions may be dangerous. Suppression of reflex swallowing by GLP-1 is thought to be helpful in keeping the ability of reflex swallowing constant together with the suppression of orexin-A. Orexins are secreted by hunger and/or hypoglycemia, and facilitate food intake [29,30,31,32,33]. Peripheral GLP-1 in L-cells is secreted just after meals [34,35]. Gastric inflation activates GLP-1-containing cells in the NTS [36,37]. Thus, meal-related factors, such as hyperglycemia or gastric inflation, reduce orexin secretion and increase GLP-1 secretion. Even when the suppressive effects of orexin-A on reflex swallowing decrease after feeding, the suppressive effects of GLP-1 increase such that the total extent of suppression of swallowing does not change before or after meals. Thus, swallowing conditions remain constant. Although meals are a great disturbance to homeostasis, suppressive responses by appetite-increasing peptides and anorectic/satiating peptides may aid in maintaining the condition of the swallowing reflex. As the swallowing threshold is maintained slightly high regardless of meals by the suppressive effects of appetite-related substances, the threshold can be lowered to promote the swallowing reflex when necessary. Mutual inhibition of orexin-A-responsive neurons and GLP-1-responsive neurons also has a similar role (Figure 5). If GLP-1 is non-functional, the inhibitory effects of GLP-1-responsive neurons on the swallowing reflex arc disappear. As the inhibitory effects on the orexin-A responsive neurons also disappear, the inhibitory effects of the orexin-A-responsive neurons on the swallowing reflex arc become conversely stronger. Therefore, the total degree of inhibition on the swallowing reflex arc is almost unchanged. The mutual inhibitory effects discovered in this study may be helpful in keeping the ability of reflex swallowing constant, even if regulatory peptides become ineffective. Thus, the living body uses many mechanisms to achieve homeostasis.

In the present study, orexin-A and GLP-1 were confirmed to have mutual inhibitory actions. Mutual inhibition between the appetite-increasing and -satiating elements is frequently reported in several physiological parameters [10,11,12,13]. Therefore, mutual inhibition between the appetite-increasing peptide and satiety peptide to alter reflex swallowing is possible. Other appetite-increasing and satiety peptides also alter the swallowing reflex [5,7,8,26]. In order to elucidate the entire mechanism of swallowing regulation by appetite-regulating substances, a comprehensive understanding, including the interaction of multiple feeding-related substances, is required.

## 4. Materials and Methods

### 4.1. Animals and Surgery

Fifty-four male Sprague–Dawley rats (280–320 g) were used. Each animal was anesthetized by intraperitoneal injection of urethane–chloralose (urethane, 0.8 g/kg; chloralose, 65 mg/kg body wt.). Subsequent anesthesia was administered through silastic tubing (outer diameter: 1.0 mm, inner diameter: 0.5 mm) inserted into the right femoral vein. Each animal had a tracheal cannula made from polyethylene tubing (OD: 2.07 mm). The sternohyoid and omohyoid muscles were retracted. The SLNs were isolated from the surrounding tissue and bilaterally sectioned near the thyroid cartilage. After the electrode was sewed onto tissue beside the nerve, the nerve end was passed and pushed into the hooks, and covered by silicone elastomer (two-part adhesive, Kwik-Cast, WPI, FL, USA).

After removing the digastric muscle, the mylohyoid muscle was exposed. A commercial bipolar recoding electrode, insulated except at the tip, (UI2-513; Unique Medical Co., Osaka, Japan), penetrated the mylohyoid muscle to record the electromyographic (EMG) activity associated with swallowing. The electrode was made of two stainless steel wires of 0.4 mm in diameter and the anode-cathode distance was 1 mm. It was not insulated 0.2 mm from the tip. Each animal was mounted on a stereotaxic apparatus. Then, the neck muscles were removed, and the ligaments between the occipital bone and the atlas were carefully removed. Part of the occipital bone and dura mater were removed, and the surface of the brainstem was exposed for drug administration. The body temperature was maintained at 36 °C using a heating pad placed under the body (ATB-1100; Nihon Kohden, Tokyo, Japan). Animal care was in accordance with the guidelines of the Physiological Society of Japan. The experimental protocols were approved by the Okayama University Animal Use Committee (approval number: OKU-2018199, approved on April 1, 2018).

### 4.2. Electrical Stimulation and Recording

The stimulating electrode was a small cuff electrode made with two 100-µm-diameter PtIr wires (Narishige. Tokyo, Japan) and epoxy resin (Devcon ET, ITW, IL, USA). One end of the 2-mm-length wires was bent in a three-quarter-circle shape in order to hook the dissected end of the SLN. The other end was connected to fine insulated lead wires by soldering. The two wire hooks were mounted in parallel in a small block of epoxy resin, in which the hook parts were exposed outside the block. The distance between the two hooks was fixed at 0.5 mm. Impedances of the electrodes measured in Ringer’s solution using a 1-V 500-Hz sine wave voltage source were 2.8–4.3 kΩ (mean; 3.5 kΩ, n = 4).

Swallowing was induced by stimulation of the central cut end of the SLN with repeat electric pulses (20 Hz, 0.2 ms in duration, 0.2 mA in intensity) for 20 s. The parameter of stimulation was similar to that in previous studies to induce reflex swallowing in anaesthetized rats [5,6,7,9,26]. Approximately 10 to 20 swallowing movements were induced during stimulation. Principally, a set of electrical pulses sustained for 20 s was delivered every 5 min except just after injection of the drug (see Figure 1A,B). Before each experimental session, the electric pulse train was delivered three times or more to confirm the stability of the responses.

The EMG signals through electrodes associated with swallowing were amplified through a biophysical amplifier with the aid of a high-cut filter (100 Hz) and low-cut filter (5 Hz), and then stored on a personal computer using LabChart software of the PowerLab system for later analysis (AD Instruments Japan Inc., Nagoya, Japan). Swallowing movements were identified under visual guidance and corresponded with EMG activity.

### 4.3. Drugs and Administration

GLP-1 (GLP-1(7-36) amide), orexin-A, SB334867, and exendin-4(5-39) (GLP-1 receptor antagonist) were used. Peptides were purchased from Peptide Institute (Osaka, Japan). SB334867 (orexin-1 receptor antagonist) was purchased from Tocris Bioscience (Bristol, UK). Peptides were dissolved in Ringer’s solution. SB334867 was dissolved in dimethyl sulfoxide (DMSO). 

For fourth ventricular administration, 3 µL of solutions was dropped around the obex using a 50-µL Hamilton syringe (Hamilton co., Reno, NV, USA). To inject the drugs into the dorsal medulla, commercial glass micropipettes (30 µm in tip diameter; World Precision Instruments Co.) connected to a 50-µL Hamilton syringe were installed on a micro injector (XF-320J; Nihon Kohden, Tokyo, Japan). Each glass pipette was first filled with paraffin fluid and the test solution (60 nL) was drawn up from the tip of a pipette. Either the mNTS or cNTS was injected into. When the mNTS was injected, the tip of the pipette was located 0.5 mm anterior to the obex, 0.3 mm lateral to the midline, and 0.4 mm ventral from the surface of the brainstem (Figure 1C). When the cNTS was injected, the tip of the pipette was located 0.5 mm anterior to the obex, just midline, and 0.6 mm ventral from the surface on the AP. The coordinates were decided according to the atlas [38] (Figure 1C). These methods for delivering drugs were similar to those used in our previous studies [2,3,5,6,9]. 

The dose of the given drugs and the procedure of administration were as follows: GLP-1 was injected into the mNTS at a dose of 6 pmol or 20 pmol. Our previous study revealed that 6 pmol of GLP-1 alone had no effect on reflex swallowing, whereas 20 pmol of GLP-1 alone suppressed reflex swallowing [9]. Orexin-A was administered into the fourth ventricle at a dose of 0.3 nmol or 1 nmol. The fourth ventricular administration of 0.3 nmol of orexin-A had no effect on reflex swallowing, whereas the fourth ventricular administration of 1.0 nmol of orexin-A suppressed reflex swallowing [6]. SB334867 was injected into the cNTS at 200 pmol to evaluate the effects of GLP-1 after blockade of orexin-1 receptors. Exendin-4(5-39) was administered into the fourth ventricle at 3 nmol to evaluate the effects of orexin-A after blockade of the GLP-1 receptors.

### 4.4. Data Analyses

The number of swallows during electrical stimulation for 20 s was counted for every stimulus event (swallowing frequency) (Figure 1B). The time between the onset of stimulation and the peak of EMG activity of the first swallow just after the onset of stimulation was measured and defined as latency (Figure 1B). All numerical values are shown as the mean ± SEM. For statistical evaluation of the measurements of swallowing repeated over time, one-way repeated measured analysis of variance (ANOVA) was used (*p* < 0.05 for significance). In general, the six time points from 5 min before administration of the drug to 25 min after were used for analysis. Dunnett’s post hoc test was used to compare between mean values 5 min before the administration of solutions and after (*p* < 0.05 for significance). As the most reliable change was obtained 10 min after the injection of GLP-1 or orexin-A according to our previous studies [6,9], the mean difference in swallowing frequency (or latency) between 5 min before administration and 10 min after was used as a representative value (Figure 1A). The two-sided unpaired *t*-test was used to compare the representative values between two groups.

## 5. Conclusions

Our previous study revealed that orexin-A, an appetite-increasing peptide, suppressed reflex swallowing induced by electrical stimulation of the SLN and that GLP-1, a satiety peptide, also suppressed reflex swallowing [6,9]. In the present study, orexin-A and GLP-1 mutually inhibited their suppressive effects on the swallowing reflex. This strongly suggests a mutual inhibitory circuit between orexin-A-responsive neurons and GLP-1-responsive neurons in the dorsal medulla, as shown in Figure 5.
